# The colors of life: an interdisciplinary artist-in-residence project to research fungal pigments as a gateway to empathy and understanding of microbial life

**DOI:** 10.1186/s40694-021-00130-7

**Published:** 2022-01-10

**Authors:** Sunanda Sharma, Vera Meyer

**Affiliations:** Chair of Applied and Molecular Microbiology, Technische Universität Berlin, Str. des 17. Juni 135, 10623 Berlin, Germany

**Keywords:** Pigmentation, Art, Interdisciplinary, *Aspergillus*, Color, Bio-art

## Abstract

**Background:**

Biological pigmentation is one of the most intriguing traits of many fungi. It holds significance to scientists, as a sign of biochemical metabolism and organism-environment interaction, and to artists, as the source of natural colors that capture the beauty of the microbial world. Furthermore, the functional roles and aesthetic appeal of biological pigmentation may be a path to inspiring human empathy for microorganisms, which is key to understanding and preserving microbial biodiversity. A project focused on cross-species empathy was initiated and conducted as part of an artist-in-residence program in 2021. The aim of this residency is to bridge the current divide between science and art through interdisciplinary practice focused on fungi.

**Results:**

The residency resulted in multiple products that are designed for artistic and scientific audiences with the central theme of biological pigmentation in fungi and other microorganisms. The first product is a video artwork that focuses on *Aspergillus niger* as a model organism that produces melanin pigment in a biosynthetic process similar to that of humans. The growth and morphology of this commonplace organism are displayed through video, photo, animation, and time-lapse footage, inviting the viewer to examine the likenesses and overlaps between humans and fungi. The second product is The Living Color Database, an online compendium of biological colors for scientists, artists, and designers. It links organisms across the tree of life, focusing on fungi, bacteria, and archaea, and the colors they express through biological pigmentation. Each pigment is represented in terms of its chemistry, its related biosynthesis, and its color expressions according to different indices: HEX, RGB, and Pantone. It is available at color.bio.

**Conclusions:**

As fungal biotechnology continues to mature into new application areas, it is as important as ever that there is human empathy for these organisms to promote the preservation and appreciation of fungal biodiversity. The products presented here provide paths for artists, scientists, and designers to understand microorganisms through the lens of color, promoting interspecies empathy through research, teaching, and practice.

“*The most important thing for humanity today and tomorrow is dialogue - dialogue between people, between I and you, in small manageable groups. For face-to-face encounters alone can bring about creative, political and cultural reflection and action*.”

Heinrich Ott, Emeritus Professor, University of Basel.

“*Art and science are in a tension that is most fruitful when these disciplines observe and penetrate each other and experience how much of the other they themselves still contain*.”

Konrad Liessmann, Professor, University of Vienna [1]

## Background

The limited discovery and quantification of microbial diversity is a significant challenge to our understanding of the biodiversity of Earth. A great deal of life on our planet may in fact be microbial, yet we are estimated to know less than 1% of existing microbial species [2] and little to nothing about the trends regarding its diversity and rate of change [3]. Even the microbial species we know of, including many bacteria, fungi, archaea, and protists, are often understudied. This may be in part due to the fact that they are individually difficult or impossible to discern with the naked eye, limiting observation and interaction by humans. The mismatch of physical scale between microorganisms and humans has been proposed as the reason for a “size bias” against microbial life, resulting in their exclusion from the ethical frameworks utilized in laboratory research [4, 5]. In addition, microorganisms lack key features that humans have been shown to have strong affective and empathetic responses to, such as visible neotenic characteristics, similarity to human appearance, the possibility of communication, and aesthetic beauty [6]. Furthermore, research on human empathy for other organisms indicates that there is an inverse relationship between empathy inspired by the species and evolutionary divergence time, suggesting that achieving human empathy for microorganisms is a challenging endeavor [7]. Yet, it is well accepted that microorganisms are essential to agriculture [8], major biogeochemical cycles [9, 10], and the evolution of higher life forms [11, 12]. In addition, they are ubiquitous in and on the human body [13] and built environment [14, 15], so may be deserving of unique ethical consideration. Microbial diversity is fundamental to not only the maintenance of global resources and, in turn, human survival [16, 17], but microorganisms are now being increasingly pursued for their potential in biotechnological applications such as the production of biopharmaceuticals [18], and use in bioremediation [19, 20]. Given that human preference directly affects the success of preservation and conservation efforts [21], it is critical that microbes are reconsidered in an empathetic light if their survival and diversity are to be maintained.

Approaches for increasing human empathy for non-human organisms have been explored most widely in the field of conservation biology and can be grouped into five themes: promoting anthropomorphism, demonstrating utility, eliciting emotion (such as sympathy, protectiveness, or curiosity), promoting practical engagement, and attachment to nature, and highlighting aesthetic beauty. The first approach focuses on finding or creating similarities between a target species and humans to develop empathy, such as by adding human-like faces onto representations of animals; it has more recently been refined in an attempt to reduce anthropocentric bias [22]. The second approach focuses on examining and communicating the usefulness of a target species to human survival or daily life. For instance, public interest in insect pollinators has been sought by presenting data on their widespread positive effect on globally important crops as well as quantifying their service contribution to market output [23]. The third approach has similarly been used to call for support for pollinators such as honeybees (*Apis mellifera*) by describing their plight and the potential role of humans as protectors [24]. The fourth approach has been explored through citizen science efforts to engage the public in the research and conservation of various organisms such as native North American songbirds [25], butterflies [26], and bumblebees [27]. Finally, highlighting aesthetic beauty has been used effectively to promote interest in some organisms, such as butterflies [28].

In the context of microorganisms, most of these approaches can be explored through the lens of biological pigmentation. Microorganisms, including certain fungi, bacteria, archaea, algae, and protists, create a stunning myriad of colors through natural pigment production. For instance, “watermelon snow”, a phenomenon found in many high altitude regions, is caused by frost-dwelling *Chlamydomonas nivalis* algae that produce the red pigment astaxanthin [29]; red fermented rice is colored by pigments from the fungus *Monascus purpureus* [30]; and decaying fruits and vegetables often have a mélange of green, brown, black, and white colors caused by pigments produced by molds and fungi such as *Penicillium*, *Mucor*, and *Aspergillus* [31]. Across taxa and environments, microorganisms are recognized for their creation of striking pigments, hundreds of which have been isolated and studied to date.

Pigments are biosynthesized through various pathways dependent on both the genetics of the organism and may be contained within cells or diffuse throughout surrounding media [32]. Most microbial pigments are termed “secondary metabolites”, as they are byproducts of biosynthesis but are not required for necessary functions such as growth and reproduction [33]. However, they are thought to have a wide variety of roles in different organisms and environments, such as photoprotection, cell wall integrity and defense, biofilm formation, and protection from oxidation [34, 35]. Extensive research has been conducted in various pigment-producing microorganisms that indicate that growth conditions and abiotic stresses, such as the presence of light [36–38], pH [39, 40], salinity [41], and temperature [40, 41], may all affect the rate and quantity of pigments produced. The many roles and diverse sources of microbial pigments have led to a significant interest in their use in several fields, including food coloration [42], textile dyeing [43–45], biomedicine [46], and synthetic biology [47, 48]. Fungal pigments, in particular, have also been used in the fields of art and design, having featured in projects such as “C-MOULD: living paints” [49, 50], in stained wood objects for a production of “The Blue Forest” [51], and in the fungi dress “Fibre Reactive” [52].

The range of applications of microbial pigments in addition to the methods and techniques required to produce and obtain them in a controlled manner naturally means that the field is inherently interdisciplinary. This is also reflected in historical approaches to cataloging biological pigmentation. For instance, the renowned *Werner’s Nomenclature of Colors*, published in 1814, is a color dictionary that contains color names matched with an animal, vegetable, and mineral source [53]. It was famously compiled by a flower painter based on the notes of a geologist, and subsequently used by evolutionary biologists, artists, natural scientists, and philosophers; more recently, it has been re-interpreted and provided as an online resource [54]. This and similar historical guides instilled a sense of wonder and interest not only in scientists but also in artists. However, few modern guides exist that capture this interdisciplinary spirit and provide accessible information for researchers of different fields, though more data than ever has been collected on pigments across domains of life.

Currently, known biological pigments produced by microorganisms are mainly cataloged in scientific publications and reviews and may be referred to in large, general databases such as Kyoto Encyclopedia of Genes and Genomes (KEGG) [55] and PubChem [56]. There also exist a handful of more specific online databases, such as ProCarDB [57], antiSMASH [58], and the *Aspergillus* secondary metabolite database [59], that include certain types of pigments but are not exhaustive. While these existing resources contain a great deal of highly pertinent information, they are often difficult to find and search within and do not provide easily comprehensible data for artists, designers, and others who are beyond the fields of microbiology or biochemistry. This hinders the transition from the collection of pigment data to the application of pigment biotechnology across fields in art, science, and society. To this end, it is clear that there is a need for a searchable, online database that is openly accessible and contains data relevant for applied researchers from science to design to art.

In this project, biological pigmentation was explored as a path for the understanding of and empathy for microorganisms, focusing on fungi. The aim of this work is to elucidate visible, attractive features of microorganisms that can promote empathy in humans, ideally encouraging the appreciation and preservation of microbial life and biodiversity. We proposed to increase empathy through the exploration of microbial color as an appealing feature in organisms that promotes sympathetic attention [60], description of the source pigments with respect to their utility and encourage practical engagement through an interactive catalog of color. This research was produced as part of an art residency in the lab Applied and Molecular Microbiology over a period of eight months. The broader motivation was to deepen both the scientific and artistic practice related to fungi and to remediate the gap between these fields through the creation of interdisciplinary research products that are released into the public realm [61]. For this reason, three complementary products and venues were chosen to display the resulting work: a video released in conjunction with an art exhibition; an open-access, searchable database hosted online; and an article written for publication in an open-access scientific journal.

## Results and discussion

### Colors of life I: a video artwork

The study of color is one of the oldest pursuits of both science and arts and has yielded distinct representations in each field, such as UV–Vis spectra and the color wheel. In biology, color is often seen as related to function—color for warning, attracting mates, camouflage, or environmental protection [62]. In the arts, color is examined mainly through human experience, emotion, and connection to visible objects and surroundings [63–65]. Pigment science and color theory are therefore two sides of the same coin and are the focus of this video artwork. This project provides a path to discover a model microbial species, *Aspergillus niger*, in a new light, expressing the visible color diversity in a single organism. In doing so, this work demonstrates a new paradigm for interdisciplinary research in which the artistic perspective does not take the role of beautification of science but aspires to engender empathy in a scientific investigation by highlighting recognizable similarities between humans and this mold.

*Aspergillus niger* is a filamentous fungus that was discovered over 100 years ago and has since taken a prominent role in the industrial production of citric acid and food enzymes [66]. Furthermore, it has been discovered in the human ocular [67] and oral [68] microbiomes, as well as in a range of built environments [69, 70], even including the International Space Station [71, 72]. Despite its ubiquity, general safety to humans [73], and positive roles in both biotechnology and human health, *Aspergillus niger* is often termed a “black mold” and is associated with rotting fruits and vegetables [74–76]. The striking color of this species is the point of interest and overlap highlighted in the video artwork, as the pigment responsible for this color, melanin, is also naturally produced by humans. This tense mixture of familiarity, disgust, and similarity makes *Aspergillus niger* an intriguing subject for this artwork. Furthermore, the selection of this melanin-producing species invites comparison to human skin color. While skin color is a highly visible component of human physical and racial identity, the juxtaposition with a distinctly non-human entity here poses both a provocation and a question regarding the “humanness” of this trait.

The video begins with a quote from Charles Darwin, the noted naturalist who utilized *Werner’s Nomenclature of Colors* in his scientific explorations [77]. The background of this research area, empathy for other species, is introduced through text and animations. The central question, “How can we learn empathy for microorganisms?” is superimposed over footage of *A. niger* conidia observed through an optical microscope. Microbial colors are presented in a format similar to the British Standard Colour Chart [78] and melanins are highlighted as a group of interest. *A. niger* is introduced in a familiar setting, on a rotting vegetable, and then as the subject of portrait time-lapses, at various magnifications, and images showing the color variation in a single culture. The metabolic pathways that produce melanin in both *Aspergillus* and humans are shown simultaneously, and examples of melanin in fungal spores and human skin are shown side by side.

**Fig. 1:**
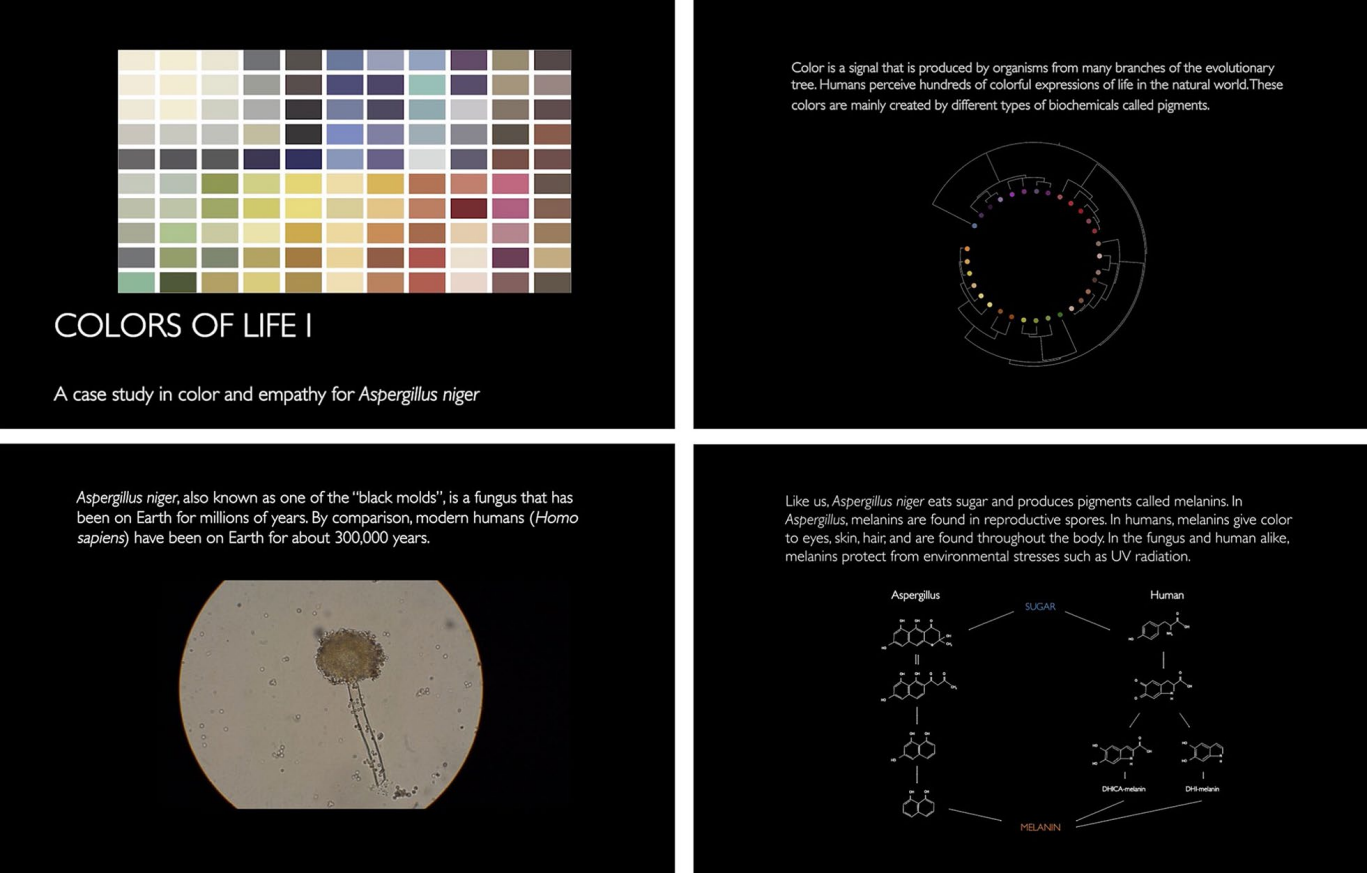
The colors of life I video. Four screenshots from the video artwork that was created as part of the residency

As non-diegetic music is commonly used to guide viewers’ emotional responses to a subject or scene [79], a novel track was composed that may evoke sympathy in the viewer, with underlying themes of repetition, mutation, and growth. It is played on a harp (as a software instrument), which has been shown to inspire emotional responses [80] and foster positive associations in humans [81]. The video is available online, in both English and German, at https://www.ssunanda.com/colors-of-life-i, on Youtube (https://youtu.be/rygnsEiIrgw, https://youtu.be/5Fvb17asoeg), and on Vimeo (https://vimeo.com/568137146, https://vimeo.com/568139990). It was released online in conjunction with the exhibition of a habitable fungal sculpture, entitled MY-CO SPACE, created by the SciArt collective MY-CO-X at tinyBE in 2021 (https://tinybe.org/artists/my-co-x/) (Fig. 1).

### Living color database: an open source artistic-scientific database

**Fig. 2:**
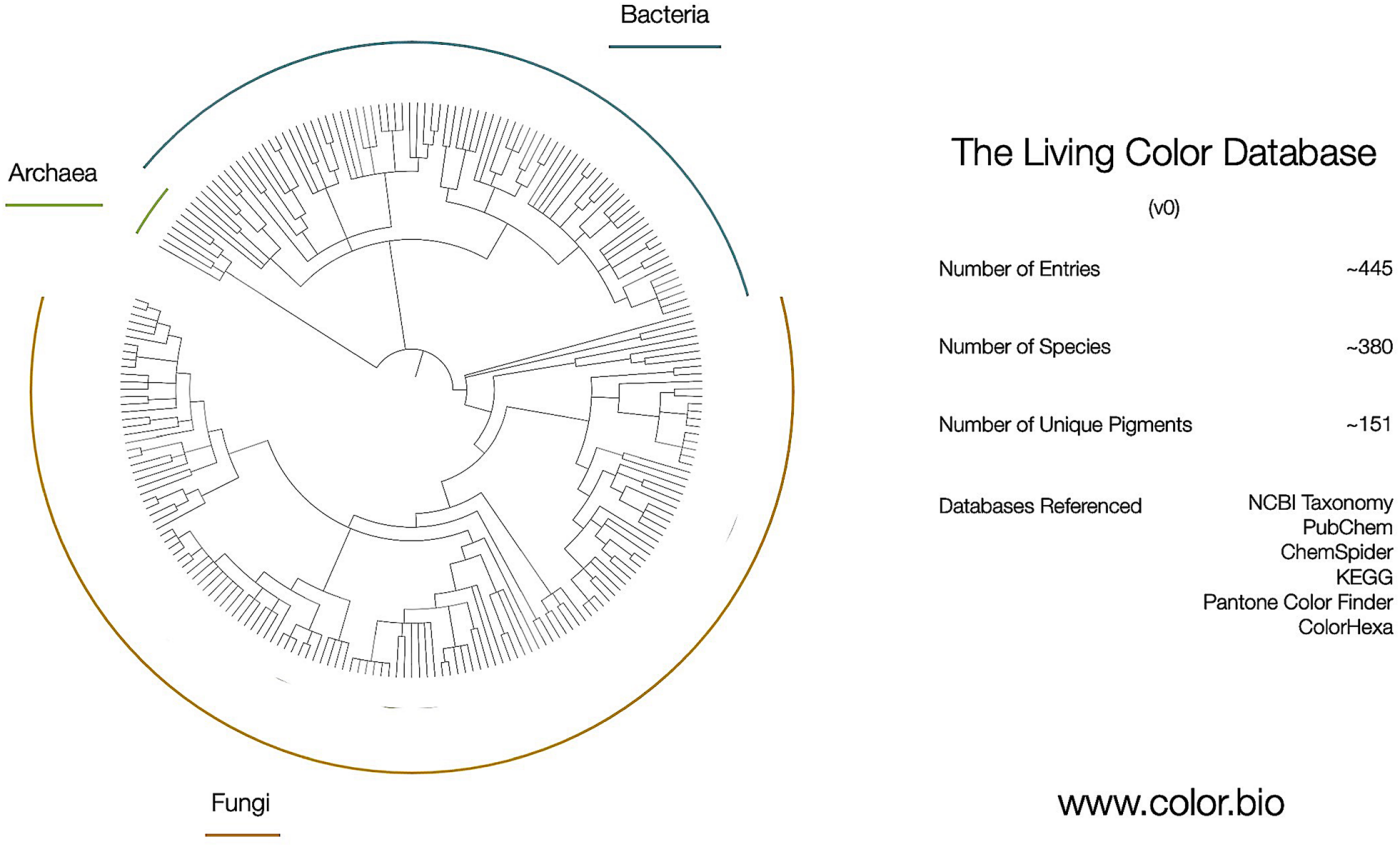
Overview of the living color database (LCDB). The LCDB includes ~ 445 entries from ~ 380 species representing ~ 151 unique pigments from fungi, archaea, and bacteria. A larger version of this figure and Newick formatted files of all species included can be accessed at www.color.bio

**Fig. 3:**
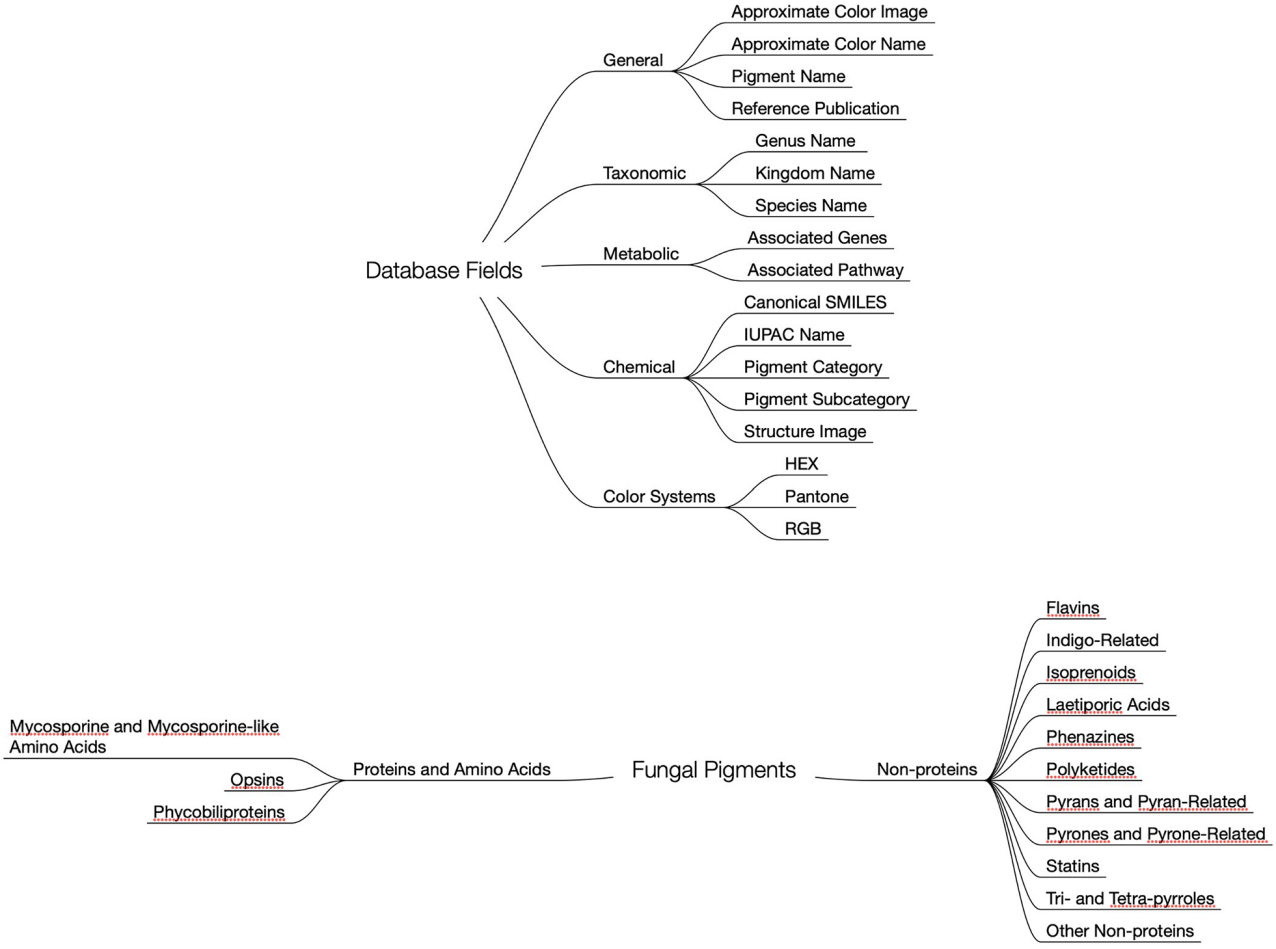
Organization of the living color database. The living color database (LCDB) includes five categories of fields to organize associated data for each entry: General Information, Taxonomic, Metabolic, Chemical, and Color Systems (top). Fungal Pigments included in the LCDB are divided by molecule type and category as organized here (bottom). Categories are further divided into subcategories (not shown)

The second product of this residency is the Living Color Database (LCDB), an online dictionary of microbial pigments that aims to correlate general pigment information with taxonomic, metabolic, chemical, and color index data (Figs. 2, 3). The initial version of this database contains approximately 445 pigment entries, representing 110 unique pigments and 380 distinct species. Currently, the database focuses on pigments from fungi and bacteria; in the future, it will be expanded to include pigments from algae and protists and contributions from other researchers across the fields of science, art, design, and engineering. The data in LCDB were sourced from published literature and existing accessible databases. Relevant keywords were used to search Google Scholar and PubMed, including: “microbial pigments”, “microorganisms pigments”, “fungal pigments”, “bacteria pigments”, “carotenoids”, “melanins”, and “prokaryotic pigments”. To allow for cross-comparison to existing databases such as ProCarDB, the following data categories were included: Pigment Name (e.g. “Aleuriaxanthin”), General Color Name (e.g. “Red”), link to source reference, Kingdom (e.g. “Fungi”), Organism Name in binomial nomenclature (e.g. “*Aleuria aurantia*”), NCBI Tax ID (e.g. “5188”), Pigment Molecule Type (e.g. “Non-protein”), Pigment Category (e.g. “Isoprenoids”), Pigment Subcategory (e.g. “Carotenoids”), IUPAC Name, Canonical SMILES, Image of Pigment Chemical Structure, Associated Genes, Relevant Publication on Organism Genetic Information or Functional Roles.

While the aforementioned data provide insights relevant for scientists from various fields, it is unapproachable for artists, designers, and application scientists who may wish to utilize biological pigmentation. Therefore, the following additional categories were included to link the organism and chemical pigment to the related visible color: Image of the Approximate Pigment Color, Approximate HEX Code, Approximate RGB values, and Pantone Color Code (e.g. “2347 C”). In the future, additional fields for images as well as evolutionary or biogeographical information for each species may be included.

The LCDB is publicly accessible on www.color.bio through any browser. The database itself is hosted on Airtable, an online, collaborative database-spreadsheet service. Airtable was selected because it allows for the creation of large databases that can be cross-correlated and linked, edited by multiple users dynamically, and embedded into other websites in several different views. Furthermore, it enables content filtering through each included field or multiple fields, allowing for users from different backgrounds to find relevant pigment information based on their own interests.

**Fig. 4:**
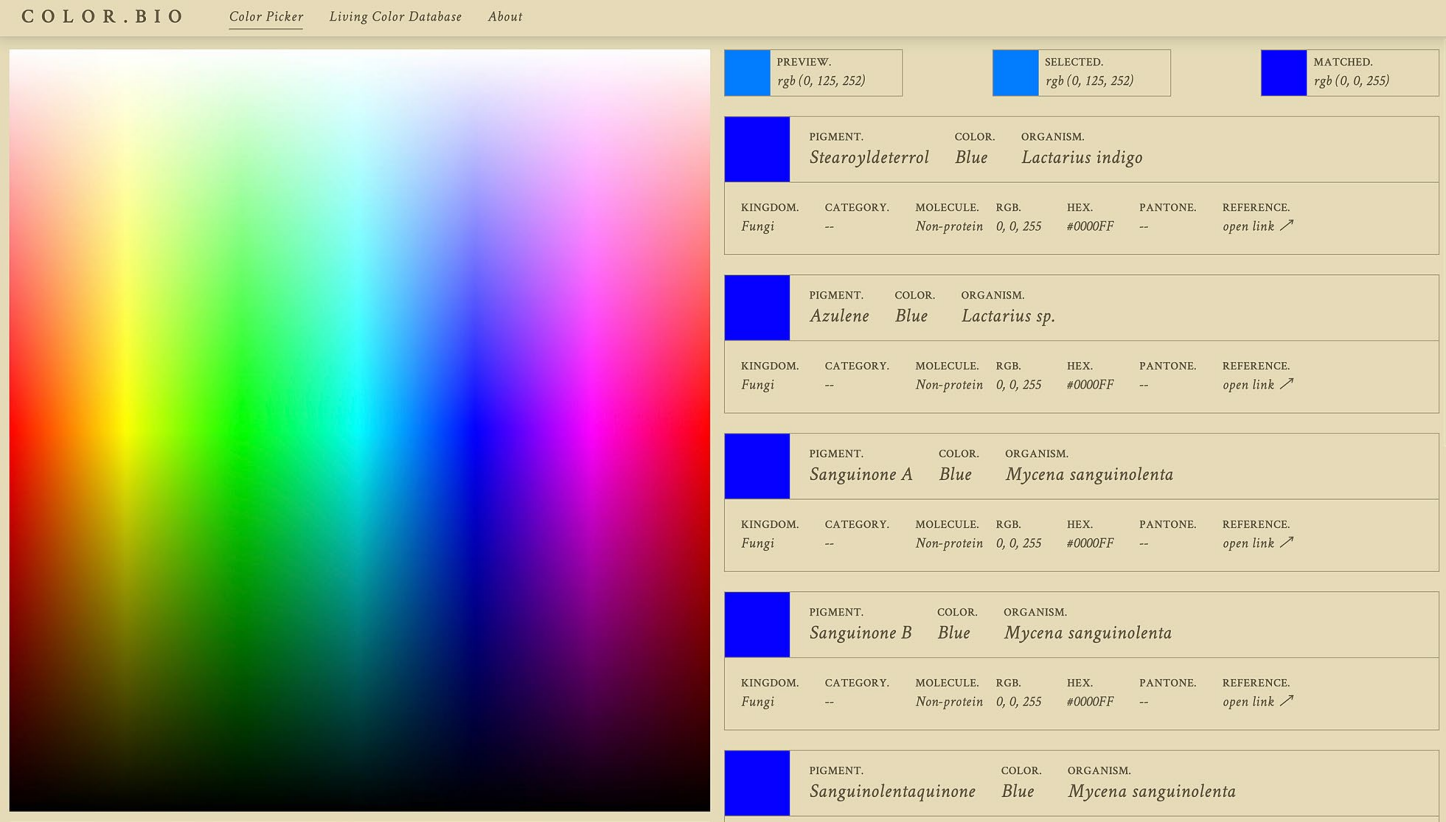
Color picker tool interface. The landing page of the www.color.bio website features the Color Picker Tool. Users may scroll over colors on the spectrum (left), view the “Preview” color, “Selected” color, and respective RGB values (top), and view the “Matched” color (top right). Cards for the closest matching pigments are returned (right) with key information, including the Pigment Name, General Color Name, Source Organism (binomial nomenclature), Kingdom, Pigment Category, Molecule Type, RGB values, HEX values, Pantone Code, and a link to a Reference

The website is organized into three pages: Color Picker, Living Color Database Entries, and About. The Color Picker allows users to select a color from an sRGB color gradient and find the closest matches to that color in terms of biological pigments in the LCDB. Data cards appear on the right that include the pigment name, approximate color, organism name, kingdom, molecule type, RGB value, HEX code, Pantone code, and link to a published reference (Fig. 4). As the LCDB is expanded, more color samples will allow for improved color matching in the Color Picker.

**Fig. 5:**
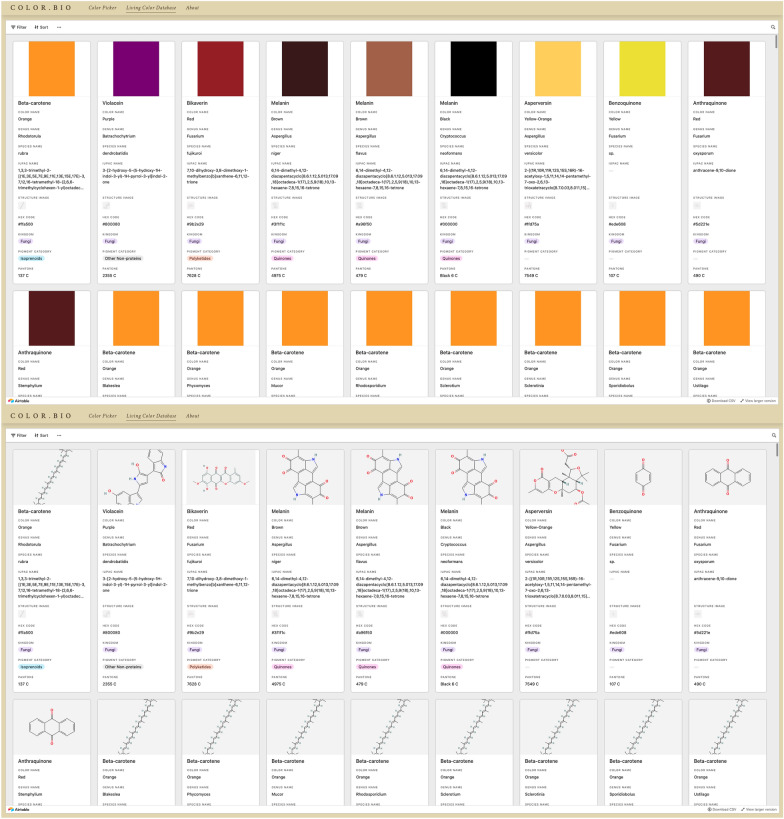
Embedded Gallery View of the LCDB. The Database can be viewed on the “Living Color Database” tab of the www.color.bio website. The card arrangement can be modified through the Airtable interface. Here, two different arrangements are shown: with an image of the Approximate Color (top) and an image of the Chemical Structure (bottom)

The Database itself is visible on the second page, where a view of the Airtable is embedded with controls for filtering and scrolling (Fig. 5). Users can see the “back end” of the database, represented in a gallery of entries, which includes all the information for each entry. In the future, there will be an interactive form to allow visitors to submit new entries to expand and update the database. The inclusion of more entries into the LCDB may allow for the identification of trends and commonalities between pigment groups and pigment-producing organisms.

## Conclusions

As increasingly more biotechnologies are being applied in new areas, multidisciplinary tools are required to expand the investigation and understanding of biology from purely scientific perspectives. These tools have the potential to not only enhance human understanding of biological phenotypes, complexity, and biodiversity but also empathy for the biological sources—the microorganisms—themselves. The aim of this residence project was twofold: to explore biological color as a path to inspiring empathy for microorganisms and catalog the vast amount of information known about microbial pigments in an accessible format for scientists, designers, and artists alike. Through the creation of three products—the video artwork, database, and research publication—this project applies multiple approaches from conservation biology to increase empathy for fungi in an attempt to appeal to the broadest possible audience. Color as an aesthetic quality is greatly featured [60]. The usefulness of pigments, and by extension, their microbial sources are highlighted in the open-access database. By utilizing language and terms from different fields, the products of the residency provide multiple parallel ways for humans to engage with organisms distant from us on the evolutionary tree.

## Materials and methods

### Colors of life I video creation and composition

Photographic and video footage was filmed using a Nikon (Melville, USA) D3300 DSLR Camera with 18–55 mm and 35 mm lenses. Micrographic footage was captured using this camera in addition to Wild M240 Makroskop and Olympus BH2 optical microscopes. Additional footage was provided by Vera Meyer and Stephan Starke (TU Berlin). All footage was edited using After Effects 2021 and compiled using Premiere 2021, both applications from Adobe Creative Cloud (San Jose, USA). Drawings and illustrations were created using Savage Interactive Procreate (Hobart, Australia) and Adobe Illustrator 2021 (San Jose, USA). Animations were created and edited using After Effects 2021. Music was composed and generated using a Yamaha P115 Electronic Keyboard (Hamamatsu, Japan) and recorded, edited, and mixed using Apple GarageBand for Mac (Cupertino, USA).

### Living color database creation

The database content, hosted on Airtable, was generated through manual entry of data sourced from published research articles, reviews, and books. Each field was arranged as a column and each pigment entry was a single row; the binomial name of each species and the associated taxonomic identification was entered in a separate sheet within the same base as a reference table. Key chemical information and imagery were sourced from the National Center for Biotechnology Information (NCBI) PubChem Database [82] and ChemSpider [83]; genetic and functional role information was sourced from KEGG and published literature, and taxonomic information was sourced from NCBI Taxonomy Browser. HEX values were identified by applying a color picker extension to images of the pigment in published literature (Hex Color Picker [84]) and subsequently converted to RGB values (ColorHexa [85]), and matched with Pantone colors (Pantone Color Finder [86]).

### Website creation

The domain www.color.bio was purchased through Google Domains (Mountain View, USA) and created with ReactJS and Tailwind CSS deployed on Vercel via GitHub. The Airtable Gallery view was selected due to its clear representation of each sample that includes both textual and visual information on a single card; this view was embedded in the “Living Color Database” tab on www.color.bio. Updates to the visible gallery on www.color.bio are periodically pushed using the Airtable API.

## Data Availability

The “Living Color Database” is available on www.color.bio.
